# It is high time we reduce “routine” blood work in neonatal units

**DOI:** 10.3389/fped.2023.1147512

**Published:** 2023-03-09

**Authors:** Michael Narvey, Minesh Khashu

**Affiliations:** ^1^ Department of Neonatology, Women's Hospital Winnipeg, University of Manitoba, Winnipeg, MB, Canada; ^2^ Neonatal Unit, University Hospitals Dorset, Poole, United Kingdom

**Keywords:** neonatal, NICU, bloods, bloodtests, testing

Painful procedures are commonplace in the neonatal intensive care unit (NICU) environment and sampling for blood tests is perhaps the most common source. Several studies have provided estimates of the frequency of hematologic testing in neonatal units. A systematic review including 18 studies examined the frequency of painful procedures in the NICU during the first 2 weeks of life and found a range of 7.5–17.3 per day (1). During an entire hospital admission, 242 stable preterm infants underwent 4,081 skin breaking procedures for an average of 17 procedures per infant. The numbers would be anticipated to be much higher for those patients who are deemed to be unstable. For those born prior to 28 weeks’ gestation, the median cumulative blood loss of the first 28 days of life from such testing was 24.2 ml/kg (IQR 15.8–30.3 ml/kg) (2). This represents almost 24%–30% of the circulating blood volume of such infants and it is therefore no surprise that infants in this same cohort received a median volume of 20 ml/kg of packet red blood cell transfusions (pRBCs) during the same period. Strategies to mitigate blood volume loss include obtaining initial bloodwork from the umbilical cord (3), deferring cord clamping (4) and if at all possible, avoiding a NICU admission altogether. The tendency to “medicalize” the newborn in the NICU, is unfortunately, still quite common and simply caring for an infant on a mother baby unit, when possible, may spare these infants unnecessary blood sampling. For example, infants admitted to a neonatal unit are more likely to be placed on a rigid expected feeding regimen and perceived failure to reach the prescribed “volume” of feeding may trigger an evaluation of electrolytes. The same infant “demand” feeding on a mother baby unit and taking only a few milliliters of colostrum who appears well, would be spared such testing.

It is clear that admission to the NICU carries with it a risk of a significant number of skin breaking procedures. One aspect of neonatal care that is pervasive in NICUs is the tendency to protocolize care. While this improves safety in certain situations, in some settings it can be detrimental to the infants if the protocol is extended to an inappropriate population group. As an example, it is not uncommon to create routine blood testing regimens in order to ensure consistency such as when starting total parenteral nutrition. This approach is sensible in terms of avoiding potentially correctable electrolyte disturbances, and is appropriate in some situations—but not in all. Where the strategy falters, is when it is applied to sampling of more stable infants who are far less likely to have significant aberrations in their bloodwork. Proponents of routine blood sampling in the neonate point to the potential risk associated with less frequent sampling such as the risk of missing abnormal values. But this must be balanced with the risks to the neonate. Across the globe, in neonatal units of all sizes, relatively stable babies will be subjected to “weekly” laboratory studies as a matter of routine practice. Although some blood tests may be required for monitoring for anemia and tailoring nutrition, many blood tests are done not because they are necessary or provide information to tailor management, but more to provide reassurance that we are “watching” the infant. In some ways it is an ineffective approach, utilized by us to monitor en masse rather than review each baby and interrogate whether a blood test in a particular baby is going to change current treatment or minimize any genuine risks. Rather than reviewing a baby and examining him/her we gather information by routine blood work. It is important to investigate our “routine” weekly blood tests and ask ourselves what proportion lead to a change in management for the baby and how can we reduce harm as well as waste in the healthcare system (5)? Anything that we do to tiny fragile infants should not happen unless it is of substantial benefit to the infant (6). Routine blood work generally is not of value to the patient, to the family or to healthcare professionals and may have detrimental effects for the infant. For example, it can generate anemia and predisposes a baby to certain risks including necrotizing enterocolitis (NEC) and possible poor neurological outcome. Any action that increases risks without adding a benefit does not make clinical sense (5, 6).

As our field continues to grow and evolve it is apparent that non-invasive testing is becoming more commonplace and with it the potential to avoid skin breaks and associated pain and its long term effects (7). In recent years the establishment of transcutaneous bilirubin measurement has replaced the need for serum bilirubin testing in many healthcare centers (8, 9). In ventilated neonates avoiding frequent arterial blood gas sampling by utilizing distal end tidal carbon dioxide sampling is another good example of non-invasive testing (10). Even in more critical patients, sampling of blood and inherent blood loss may be minimized through newer non-invasive means such as continuous hemoglobin measurement, which could soon become commonplace in conjunction with systemic oxygen saturation monitoring. Lastly, although preterm infants experience a greater number of skin breaks than the average term infant, newborns with hypoglycemia are often exposed to many skin breaks a day from repeated point of care glucose measurements. Recent literature has demonstrated that continuous glucose measurement through subcutaneously implanted devices is both accurate and can help reduce such testing to a couple occurrences a day for calibration in both the preterm and term populations with hypoglycemia (11, 12).

Despite advocating strongly for the use of non-invasive technology there are clearly times when blood sampling is required. It is incumbent on healthcare providers to consider when it is needed and to adopt strategies to minimize blood work by considering all the information available from a test. When a blood gas is drawn for respiratory management, one can also obtain values for the commonly tested electrolytes that might otherwise be sent to a laboratory as a separate sample. Using electrolytes obtained from a blood gas sample may not correlate exactly to the serum samples sent to the laboratory but are helpful in terms of monitoring trends and deciding the need for a laboratory test. Every drop of blood should be treated as precious and obtaining all the information one can from an individual test may decrease our reliance on repeated sampling and further leads us to critically think about every blood test being requested.

If we want to improve our systems, we need to think critically about every patient every time. By applying the practice of routine blood work, are we abdicating “thinking” with respect to the particular patient's current condition. Moreover, when we don't order sampling with intention, we put clinical care at risk by being less aware that there is a test that needs follow-up.

How does the clinician then affect meaningful change without compromising safety? Proponents of routine bloodwork often cite the reduced likelihood of missing an important derangement in need of attention. All over the globe in one form or another, neonatal teams carry out rounds at least twice a day and make decisions on care. We propose a model of rounding that ends each patient report with the following question. *Are there any blood tests that should be performed in the next 12–24 h*? By making this question explicitly an important part of rounds each team would need to think critically about the benefits and best timing of each test. Moreover, the biggest area of unnecessary blood testing is within the less intensive part of neonatal units, the so-called “feeders and growers” who may be subjected to weekly blood testing that may not at all be required (3). Some may point out that this deimplementation can't be supported by evidence at this stage as that research evidence does not exist. In our opinion these weekly testing regimens in biologically stable infants were not borne out of research or high quality evidence. We would welcome further research in this domain. This call for further evidence should, however, not prevent us from taking some pragmatic actions now. All we are asking of clinicians is to “think” before doing a so-called routine blood test and go through a 3-step process to justify the blood test:

1. Has the infant been examined prior to the “routine blood test”? (In most units this is a combination of Full Blood Counts, electrolytes and renal function tests +/− liver function and calcium profile tests)
2. When were the last tests done, what were they and is a repeat in a week justified?
3. Based on the examination of the infant, the medications and stability of its biological systems how likely is a derangement of the “routine tests” expected which would necessitate a change in management of the infant. These seemingly “simple” steps could help reduce unnecessary blood sampling and along with other measures like delayed cord clamping alluded to above, could significantly decrease the risk of iatrogenic anemia and its potential complications.

We owe it to our patients to provide the best care possible. However, providing the best care doesn't necessarily equate to more testing. Prevention of disease and iatrogenic complications are hallmarks of safe and effective healthcare and in the context of the neonate, preventing anemia and minimising blood testing are high priorities.
